# Adipose Tissue Aging and Natural Interventions: Potential Roles of Polyphenols and Polysaccharides

**DOI:** 10.3390/nu18060927

**Published:** 2026-03-15

**Authors:** Zhao-Jie Chen, Zi-Yan Zhao, Yi-Yi Chen, Zhen-Chi Li, Yong-Xian Cheng

**Affiliations:** 1 School of Traditional Chinese Medicine, Southern Medical University, Guangzhou 510515, China; m13420105143@163.com; 2 School of Pharmacy, Shenzhen University Medical School, Shenzhen University, Shenzhen 518055, China; 2410245062@mails.szu.edu.cn (Z.-Y.Z.); cyy03021024@163.com (Y.-Y.C.); 3 School of Pharmacy, Guangdong Pharmaceutical University, Guangzhou 510006, China

**Keywords:** adipose tissue aging, natural products, polyphenols, polysaccharides, anti-aging

## Abstract

Adipose tissue serves as a critical metabolic and endocrine organ, essential for maintaining systemic energy homeostasis and inter-organ communication. During the aging process, it undergoes significant structural remodeling and functional decline, characterized by dysregulated lipid metabolism, chronic low-grade inflammation, reduced insulin sensitivity, and adipokine imbalance. These alterations not only compromise the physiological integrity of adipose tissue but also contribute to the progression of various age-associated metabolic disorders, including type 2 diabetes, atherosclerosis, and nonalcoholic fatty liver disease. In recent years, natural products have emerged as a focal point in anti-aging research, owing to their broad accessibility, high biological safety, and capacity for multi-target regulation. Polyphenolic and polysaccharide, in particular, have demonstrated robust antioxidant, anti-inflammatory, autophagy-modulating, and mitochondrial-protective effects in cellular and animal models, indicating their promise in attenuating adipose tissue aging. Although the anti-aging effects of these natural compounds are well documented in the neural, hepatic, and cardiovascular systems, their specific mechanisms in adipose depots—especially differential regulatory patterns between white and brown adipose tissues, which may inform depot-specific therapies—and the development of targeted delivery approaches remain inadequately explored. This review, grounded in the three primary hallmarks of adipose tissue aging (oxidative stress, chronic inflammation, and dysregulated lipid metabolism), systematically elucidates the molecular mechanisms and recent advancements in the application of polyphenols and polysaccharides as natural modulators. This review establishes a cohesive theoretical foundation and delivers innovative perspectives to guide the advancement of natural product-based nutritional and therapeutic strategies for combating adipose tissue aging.

## 1. Introduction

Aging constitutes a complex biological process involving progressive cellular and tissue deterioration, disruption of physiological homeostasis, and the accumulation of chronic low-grade inflammation [1,2]. As global population aging intensifies, the prevalence of age-related chronic diseases—such as type 2 diabetes, cardiovascular disorders, and metabolic syndrome—has risen sharply, posing substantial challenge to public health systems worldwide [3,4]. Contemporary aging research has evolved from describing its pathology to mechanistic elucidation, revealing interconnected pathways involving inflammation, oxidative stress, mitochondrial dysfunction, and epigenetic modification that perpetuate degenerative states and hasten disease onset [1,5]. Within this framework, adipose tissue dysfunction emerges as a central, yet underexplored, driver of systemic aging.

Beyond serving as an energy reservoir, adipose tissue functions as a crucial endocrine and immune regulatory center [6,7]. Age-related changes in adipose tissue include structural remodeling, impaired lipolysis, reduced adipogenic potential of precursor cells, increased chronic inflammation, and mitochondrial dysfunction [8,9]. These alterations promote aberrant secretion of adipokines and proinflammatory mediators, exacerbating inflammation, insulin resistance, and metabolic disturbances, thereby forming a vicious cycle that accelerates organismal aging [10,11,12]. Epidemiological data link visceral adiposity to increased all-cause mortality [13,14,15], while adipose tissue communicates bidirectionally with other organs—such as skeletal muscle, liver, and brain—via adipokines, cytokines, and exosome, coordinating multi-organ decline [16,17,18]. Therefore, elucidating adipose tissue aging mechanisms is essential for comprehending systemic aging networks and developing targeted interventions.

Current strategies to mitigate aging, including caloric restriction, exercise, and pharmacotherapies like metformin or rapamycin, partially restore adipose function by curbing inflammation and enhancing metabolism [19,20]. However, these approaches are constrained by side effects, limited specificity, and suboptimal long-term efficacy. Natural products present compelling alternative, given their accessibility, safety, and pleiotropic actions [21,22,23]. While diverse classes—such as terpenoids (e.g., celastrol, tanshinones) [24,25,26], alkaloids (e.g., berberine, evodiamine) [27,28], and saponins (e.g., ginsenosides, notoginsenosides) [29,30]—exhibit anti-aging potential through autophagy modulation, inflammation suppression, and metabolic regulation, research on their adipose-specific effects remains fragmented [31].

Among natural compounds, polyphenols (e.g., resveratrol, quercetin) and polysaccharides (e.g., from *Astragalus* and *Ganoderma lucidum*) stand out for their validated antioxidant, anti-inflammatory, lipid-regulatory, and mitochondrial-protective effects in cellular and animal models [32,33,34,35]. These have been extensively studied in neural [36,37], hepatic [37,38], and cardiovascular [39] aging contexts, but systematic evaluations in adipose tissue—particularly integrating mechanisms like oxidative stress, inflammaging, and lipid dysregulation—are lacking. This review focuses on these hallmarks, synthesizing molecular pathways through which polyphenols and polysaccharides modulate adipose aging. By outlining recent advances and proposing integrated frameworks, it aims to bridge mechanistic insights with translational strategies, fostering novel nutritional and therapeutic approaches to delay adipose tissue senescence and its associated pathologies.

## 2. Materials and Methods

The present work was conducted as a narrative review. Relevant literature was collected from PubMed, Web of Science, and Scopus using keyword combinations related to adipose tissue aging and natural products, including “adipose tissue aging”, “adipose senescence”, “polyphenols”, “polysaccharides”, “oxidative stress”, “inflammation”, and “lipid metabolism”. Studies were selected based on their relevance to the role of polyphenols and polysaccharides in the regulation of adipose tissue aging, with particular attention to mechanisms involving oxidative stress, chronic inflammation, and lipid metabolic dysfunction. Both original research articles and representative review articles were considered. The included studies were subsequently organized according to the major pathological features of adipose tissue aging and the corresponding mechanistic actions of natural compounds. Figures were created using BioRender (web-based platform; BioRender, Toronto, ON, Canada)

## 3. Adipose Tissue Aging: Hallmarks and Systemic Impact

### 3.1. Structure and Functional Characteristics of Adipose Depots

Adipose tissue is a metabolically active endocrine organ composed primarily of white adipose tissue (WAT) and brown adipose tissue (BAT). WAT functions not only as the major energy storage depot through triglyceride accumulation and mobilization but also as an endocrine organ that secretes adipokines such as leptin, adiponectin, and resistin, thereby participating in the regulation of systemic metabolic homeostasis [40]. Based on anatomical location, WAT is further classified into visceral adipose tissue (VAT) and subcutaneous adipose tissue (sWAT), which exhibit distinct structural characteristics, metabolic properties, and aging-associated alterations [41]. During aging, VAT becomes increasingly susceptible to inflammatory cell infiltration, excessive lipolysis, and reduced insulin sensitivity, resulting in heightened secretion of pro-inflammatory cytokines and the accumulation of lipotoxic intermediates. These changes significantly increase the risk of insulin resistance, type 2 diabetes, and cardiovascular disease [40,42]. In contrast, sWAT, which exhibits higher insulin sensitivity and stronger metabolic protective capacity in youth, gradually loses its ability for healthy expansion with age due to impaired preadipocyte differentiation, increased fibrosis, and reduced lipid-buffering capacity. These alterations promote ectopic lipid deposition and systemic metabolic dysregulation [43]. Therefore, VAT and sWAT contribute to metabolic dysfunction through distinct mechanisms: VAT via excessive inflammatory activation and lipotoxicity, and sWAT via reduced metabolic flexibility and protective buffering capacity, collectively exacerbating systemic metabolic instability during aging.

BAT, in contrast to WAT, specializes in non-shivering thermogenesis through uncoupling protein 1 (UCP1)-mediated mitochondrial respiratory uncoupling, thereby maintaining body temperature and promoting energy expenditure [40,44]. However, aging leads to a marked decline in BAT activity, mass, and UCP1 expression, accompanied by mitochondrial dysfunction and impaired thermogenic capacity [45]. Additionally, the secretion and bioactivity of batokines, such as FGF21, 12,13-diHOME, and exosomal non-coding RNAs, progressively decrease with age, reducing BAT’s ability to regulate glucose homeostasis, lipid metabolism, and insulin sensitivity [45,46]. Structural aging of BAT also involves decreased vascularization and diminished sympathetic innervation, further limiting its thermogenic responsiveness and metabolic protective capacity [47]. Consequently, BAT aging plays a pivotal role in the progressive decline of systemic energy expenditure and metabolic homeostasis.

### 3.2. Core Hallmarks of Adipose Tissue Aging

Adipose tissue aging is fundamentally characterized by three interrelated pathological hallmarks: oxidative stress, chronic low-grade inflammation, and dysregulated lipid metabolism. Rather than acting independently, these processes are tightly interconnected and collectively form a self-reinforcing degenerative cycle. Oxidative stress not only induces DNA damage but also promotes the secretion of senescence-associated secretory phenotype (SASP) factors, thereby initiating and amplifying inflammatory signaling cascades. The resulting inflammatory milieu further impairs metabolic regulatory pathways, aggravating adipocyte dysfunction, lipid accumulation, and insulin resistance. Meanwhile, excessive lipid deposition and lipotoxicity disrupt mitochondrial integrity and function, generating additional reactive oxygen species (ROS) and further intensifying oxidative stress [48,49]. This reciprocal interplay forms a pathogenic loop that progressively accelerates adipose tissue senescence and systemic metabolic deterioration.

Notably, experimental evidence—such as the observation that antioxidant interventions can simultaneously attenuate inflammation and improve lipid metabolism—supports the existence of crosstalk among these pathways. However, the precise molecular connections, temporal sequence of their interactions, and depot-specific variations remain insufficiently understood. These unresolved questions highlight the need for integrative multi-omics approaches and targeted mechanistic studies to delineate the coordinated regulatory network underlying adipose tissue aging.

#### 3.2.1. Oxidative Stress and Mitochondrial Dysfunction

Oxidative stress is widely recognized as a major pathogenic driver of adipose tissue aging, but its underlying mechanisms and pathophysiological outcomes display depot-specific differences between WAT and BAT. With advancing age, both tissues exhibit excessive accumulation of ROS, although the sources, regulatory mechanisms, and biological consequences of ROS vary considerably [31,50]. In WAT, oxidative stress is primarily associated with a decline in antioxidant defense capacity, including reduced activity of superoxide dismutase (SOD) and glutathione peroxidase (GPx). VAT, characterized by higher metabolic activity and enriched immune cell infiltration, exhibits particularly elevated ROS levels compared to sWAT, rendering it more susceptible to DNA damage, lipid peroxidation, and protein dysfunction. These alterations suppress the adipogenic differentiation capacity of adipose-derived stem cells (ASCs), which reside in the stromal vascular fraction and are responsible for adipocyte replacement and metabolic homeostasis [31,51,52]. Under sustained oxidative stress, ASCs undergo telomere shortening, p53/p21 pathway activation, and G1 phase arrest, collectively resulting in cellular senescence, loss of self-renewal, and impaired regenerative capacity [53,54,55]. In BAT, mitochondrial dysfunction represents the principal source of ROS. Aging-associated reductions in UCP1 activity and impaired mitochondrial respiration lead to excessive ROS generation, which further exacerbates mitochondrial damage [55,56]. The decline in thermogenic efficiency, accompanied by impaired mitophagy, creates a vicious cycle that accelerates lipid accumulation and promotes BAT whitening [45,57]. In addition, oxidative stress disrupts key regulatory pathways, including AMPK, SIRT1, and Nrf2 signaling, thereby weakening antioxidant defenses, diminishing insulin sensitivity, and amplifying inflammatory responses [58]. Notably, WAT aging is predominantly characterized by oxidative damage–induced inflammation and immune cell recruitment, whereas BAT aging manifests primarily as metabolic impairments, mitochondrial dysfunction, and thermogenic decline [31]. Therefore, oxidative stress not only acts as a central trigger of adipose tissue aging but also drives depot-specific pathological remodeling through distinct molecular pathways and microenvironmental features [59].

#### 3.2.2. Inflammaging

Chronic inflammation, often called ‘inflammaging’, is a defining hallmark of adipose tissue aging and exhibits marked depot-specific heterogeneity between WAT and BAT. In WAT—particularly in VAT—aging leads to the accumulation of senescent cells that secrete pro-inflammatory cytokines including TNF-α, IL-6, IL-1β, and MCP-1 through the SASP [31,60,61]. These inflammatory mediators activate NF-κB and JAK/STAT pathways, driving macrophage recruitment and polarization toward the pro-inflammatory M1 phenotype, thereby establishing a persistent inflammatory microenvironment [62]. In contrast, BAT also undergoes inflammatory activation during aging, but the magnitude and consequences differ. In BAT, inflammation is more strongly linked to impaired UCP1 function, mitochondrial dysfunction, and immune cell remodeling, rather than overt cytokine overproduction [47,63]. Moreover, the inflammatory secretory profile of adipose tissue differs significantly by depot. In WAT—especially VAT—aging is associated with decreased adiponectin and increased secretion of leptin and resistin, thereby exacerbating insulin resistance and metabolic dysregulation [14,40]. In contrast, BAT inflammation more prominently affects metabolic regulatory factors such as FGF21, IL-6, and interferon-related cytokines, which regulate thermogenic function and systemic energy metabolism [64,65]. These distinct inflammatory characteristics lead to diverging functional consequences across fat depots. In WAT, inflammaging impairs adipogenic differentiation, promotes extracellular matrix (ECM) deposition and fibrosis, and aggravates insulin resistance. Conversely, in BAT, inflammatory signaling predominantly disrupts mitochondrial biogenesis and thermogenic capacity, thereby accelerating BAT whitening and energy dysfunction. Experimental evidence—including natural aging and D-galactose–induced models—indicates that VAT exhibits the highest degree of inflammatory activation, with persistent NF-κB signaling serving as a major driver of WAT aging [31,62,66]. Conversely, inflammation in BAT is more closely associated with the attenuation of the PPARγ/PGC-1α axis, leading to impaired thermogenesis and metabolic decline [47,63]. Collectively, these depot-specific inflammatory patterns not only exacerbate local tissue dysfunction but also propagate systemic metabolic dysregulation through endocrine and paracrine signaling, thereby further accelerating organismal aging.

#### 3.2.3. Dysregulated Lipid Metabolism and Adipokine Secretion

Dysregulated lipid metabolism represents another core hallmark of adipose tissue aging, manifesting distinct depot-specific features between WAT and BAT. In WAT—particularly VAT—aging is characterized by impaired lipolysis, reduced responsiveness to catecholamine-mediated β-adrenergic signaling, and diminished fatty acid mobilization and oxidation, leading to excessive lipid retention and ectopic deposition in peripheral tissues [45,60]. In contrast, metabolic dysfunction in BAT is primarily associated with weakened UCP1-mediated thermogenic lipolysis and decreased mitochondrial β-oxidation, resulting in impaired triglyceride utilization and reduced energy expenditure [45]. Aging also leads to substantial alterations in adipokine secretion profiles. In WAT, levels of adiponectin decrease significantly, while leptin secretion becomes dysregulated, promoting systemic insulin resistance and leptin resistance [14,40,60]. In contrast, BAT dysfunction is characterized by reduced secretion of key metabolic regulatory batokines, such as FGF21 and IL-6, which play essential roles in systemic glucose homeostasis, lipid mobilization, and thermogenic regulation [65,66]. These endocrine alterations, combined with the redistribution of adipose depots—particularly the decline in sWAT, expansion of VAT, and progressive whitening of BAT—collectively contribute to systemic lipotoxicity and the onset of age-related metabolic syndrome [14,40,60]. At the molecular level, suppressed activity of PPARγ and its downstream lipid metabolic genes, including *aP2* and *LPL*, leads to diminished lipogenic capacity and impaired lipid storage in WAT [67]. Meanwhile, attenuation of the PPARγ/PGC-1α axis in BAT disrupts mitochondrial biogenesis and thermogenic gene expression, thereby aggravating functional decline and whitening conversion [68]. These signaling disruptions highlight depot-specific vulnerabilities in lipid metabolism during aging. Although adiponectin is widely used as a biomarker of adipose dysfunction, its clinical relevance varies across fat depots. In WAT, reduced adiponectin is strongly correlated with insulin resistance and cardiovascular risk and is commonly applied as an indicator of visceral adiposity and metabolic health [69,70]. Conversely, changes in BAT-derived factors, particularly FGF21, more accurately reflect thermogenic impairment and age-related decline in energy expenditure, serving as emerging biomarkers of BAT functional aging [71,72]. Therefore, although adipokines serve as valuable biomarkers of adipose tissue aging, their interpretation must consider depot-specific secretion profiles, metabolic functions, and underlying signaling mechanisms.

### 3.3. Health Risks Induced by Adipose Tissue Aging

Adipose tissue is a central regulator of systemic metabolic homeostasis, and its age-related decline contributes to the onset and progression of various chronic diseases. The three core hallmarks of adipose tissue aging—oxidative stress, inflammaging, and dysregulated lipid metabolism—induce profound structural remodeling and functional impairment, which collectively drive metabolic, cardiovascular, neurodegenerative, and musculoskeletal disorders through endocrine, paracrine, and immunometabolic mechanisms [8,9]. The following sections outline the mechanistic contributions of aging adipose depots to major chronic disease categories.

The development of type 2 diabetes (T2D) is closely associated with adipose tissue aging, particularly the functional deterioration of VAT. Aging induces heightened lipolytic activity and attenuated catecholamine responsiveness, leading to excessive release of free fatty acids (FFAs) into circulation. These FFAs accumulate ectopically in the liver and skeletal muscle, where they impair insulin signaling, contribute to mitochondrial dysfunction, and ultimately promote systemic insulin resistance (IR) [73]. Moreover, elevated FFAs enhance hepatic gluconeogenesis and VLDL synthesis, reinforcing a metabolic cycle that links hyperglycemia with lipid dyshomeostasis—an effect consistent with the concepts of lipotoxicity and the dual-cycle hypothesis [74]. Over time, these interactions impair pancreatic β-cell function, reduce insulin secretion, and perpetuate glucose intolerance, thereby accelerating the onset and progression of T2D.

Similarly, aging of cardiac and perivascular adipose tissue plays a direct role in cardiovascular decline. Epicardial adipose tissue (EpAT), a subtype of WAT, exhibits intensified oxidative stress and secretes higher levels of pro-inflammatory adipokines such as leptin and resistin. These mediators interfere with calcium handling and electrical conduction in cardiomyocytes, increasing vulnerability to cardiac arrhythmias [75]. Meanwhile, senescent perivascular adipose tissue (PVAT) loses its vasoprotective, anti-inflammatory phenotype, shifting toward a pro-oxidative state. This change disrupts the nitric oxide (NO)/endothelin-1 (ET-1) balance, impairs endothelial relaxation, and contributes to vascular stiffening, hypertension, and atherosclerosis [76,77]

In the central nervous system, aging adipose tissue contributes to neurodegenerative pathology via inflammatory and metabolic signaling. Cytokines (e.g., TNF-α, IL-6) and adipokines (e.g., leptin, resistin) released from aging VAT can cross the blood–brain barrier and activate microglia, thereby inducing neuroinflammation, oxidative injury, amyloid-β (Aβ) deposition, and tau hyperphosphorylation—hallmark features of Alzheimer’s disease [78,79]. Similar immunometabolic signaling has been implicated in the loss of dopaminergic neurons in Parkinson’s disease, suggesting a shared inflammatory basis across neurodegenerative disorders [80].

Adipose tissue aging also indirectly contributes to osteoarthritis (OA) through adipokine-mediated disruption of joint homeostasis. Circulating leptin and resistin act on chondrocytes and synovial fibroblasts, inducing the expression of matrix metalloproteinases (MMPs) and local inflammatory mediators, which promote cartilage degradation, synovial inflammation, and OA progression [81].

Adipose tissue aging may also contribute to infertility through endocrine, inflammatory, and metabolic mechanisms. Adipose tissue regulates reproductive function by secreting adipokines such as leptin and adiponectin, which influence hypothalamic–pituitary–gonadal axis activity, ovarian steroidogenesis, follicular development, and endometrial receptivity [82]. During aging, adipose tissue senescence is accompanied by chronic low-grade inflammation, altered adipokine secretion, and systemic insulin resistance, all of which may disrupt reproductive hormone homeostasis and impair oocyte quality and ovulatory function [83]. In addition, senescence-associated inflammatory mediators may exacerbate ovarian aging and reduce reproductive capacity [84].

**Figure 1:**
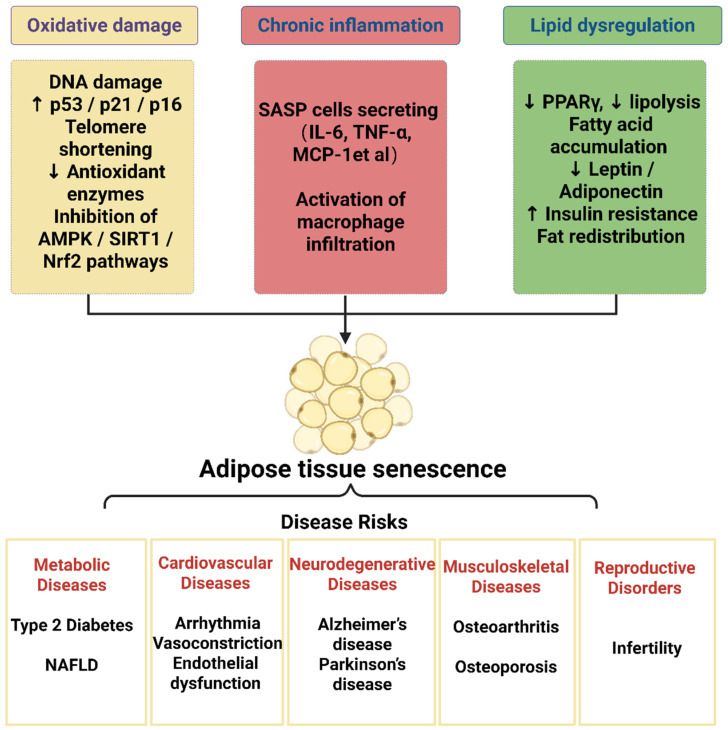
Roadmap of Adipose Tissue Aging and Related Diseases. Aging adipose tissue is characterized by three interrelated hallmarks—oxidative stress, chronic low-grade inflammation, and dysregulated lipid metabolism. These processes drive structural remodeling and functional decline of adipose depots, leading to impaired adipokine secretion, mitochondrial dysfunction, and insulin resistance. Through endocrine, inflammatory, and metabolic signaling, aging adipose tissue contributes to the development of type 2 diabetes, cardiovascular disease, neurodegeneration, osteoarthritis and infertility. This schematic summarizes how local adipose senescence propagates systemic aging and chronic disease progression. Upward arrows indicate increase/upregulation, whereas downward arrows indicate decrease/downregulation.

In summary, adipose tissue aging is not merely a localized degenerative process but a systemic pathogenic driver that, through amplified oxidative stress, chronic inflammation, and metabolic dysregulation, contributes to the development of T2D, cardiovascular disease, neurodegenerative disorders, osteoarthritis and infertility (Figure 1). From a clinical perspective, interventions targeting adipose tissue–specific aging mechanisms—such as restoring adipokine balance, enhancing antioxidant capacity, and suppressing inflammatory signaling—may offer promising strategies to delay systemic aging and prevent multiple chronic diseases, highlighting their potential in translational medicine.

## 4. Limitations of Current Interventions Targeting Adipose Tissue Aging

Despite significant progress in understanding adipose tissue aging, effective and targeted interventions remain largely limited. Most current treatment strategies focus on alleviating metabolic symptoms rather than addressing the fundamental mechanisms of adipose tissue senescence. Moreover, issues such as limited tissue specificity, inadequate efficacy, and safety concerns restrict their broader clinical applicability.

Several commonly used metabolic drugs, including thiazolidinediones, statins, and glucagon-like peptide-1 receptor agonists (GLP-1 RAs), have demonstrated partial benefits in improving adipose tissue function by reducing inflammation and enhancing glucose and lipid metabolism [85,86,87,88,89]. However, these agents were not originally designed to target cellular aging. They mainly modulate downstream metabolic pathways and therefore fail to reverse essential senescence-related processes such as oxidative damage, mitochondrial dysfunction, and epigenetic dysregulation. Furthermore, their long-term use is often associated with adverse effects, including weight gain, myopathy, and gastrointestinal symptoms, which limit their safety and clinical feasibility [90,91].

Senolytic drugs represent a more mechanism-driven approach, as they selectively eliminate senescent cells and attenuate SASP-associated inflammation. Although promising in preclinical studies, their tissue specificity, risk of off-target toxicity, and long-term safety profiles remain uncertain and require further validation through large-scale clinical trials [92,93]. Similarly, caloric restriction (CR) has been recognized as one of the most effective interventions for delaying age-related metabolic decline. However, its long-term application in humans is hindered by low adherence, high individual variability, and potential risks such as malnutrition and loss of lean body mass, which significantly limit its clinical utility [94].

In summary, existing intervention strategies struggle to safely and effectively reverse the core biological drivers of adipose tissue aging. There is an urgent need to develop multi-target, low-toxicity, and metabolically adaptable approaches that can mimic the beneficial effects of CR, modulate key aging pathways, and selectively restore adipose tissue function [95]. These limitations have fostered an increasingly strong interest in the role of natural products in combating adipose tissue aging.

## 5. Polyphenols as Modulators of Adipose Tissue Aging

Polyphenols are widely distributed in various plants, particularly concentrated in the outer skins of fruits and vegetables, such as quercetin, epigallocatechin gallate, and resveratrol. In studies focusing on adipose tissue aging, polyphenolic natural products have attracted increasing attention owing to their potent abilities to modulate oxidative stress, alleviate chronic inflammation, and improve lipid metabolism. Consequently, they have become some of the most actively investigated candidate molecules in recent anti-aging research targeting adipose tissue. To provide a clearer overview of their major sources, biological activities, and mechanisms related to adipose tissue aging, representative polyphenols are summarized in Table 1.

### 5.1. Mitigation of Oxidative Stress

Polyphenolic compounds are widely recognized for their strong antioxidant properties and have shown considerable potential in delaying adipose tissue aging. Their antioxidant actions can be broadly categorized into two mechanisms: direct chemical scavenging of ROS and activation of endogenous antioxidant defense systems by modulating key intracellular antioxidant signaling pathways. Rather than relying solely on redox chemical reactions, polyphenols exert more sustained and biologically relevant effects by modulating major antioxidant signaling pathways, particularly the Keap1–Nrf2 and SIRT1 signaling axes. The Keap1–Nrf2 pathway is a central regulatory system of oxidative stress resistance. Under oxidative stimulation or activation by polyphenols, Nrf2 dissociates from Keap1, translocates into the nucleus, and induces the transcription of antioxidant genes such as *SOD*, *CAT*, and *HO-1*, thereby enhancing cellular resilience to oxidative damage [96]. SIRT1, a key NAD^+^-dependent deacetylase, not only activates Nrf2 through deacetylation but also improves mitochondrial function, promotes mitophagy, and suppresses SASP-associated inflammation, thus helping maintain redox homeostasis at both cellular and tissue levels.

Representative polyphenolic compounds further illustrate these antioxidant effects in adipose tissue. For example, quercetin markedly increases the expression of SOD, CAT, and HO-1, reduces ROS and MDA accumulation, and attenuates senescence in both preadipocytes and mature adipocytes [96]. EGCG enhances antioxidant enzyme activity and improves mitochondrial biogenesis and function, thereby delaying adipose tissue degeneration [115]. Similarly, resveratrol facilitates ROS clearance and improves mitochondrial homeostasis, while curcumin promotes antioxidant enzyme activity and alleviates age-related oxidative damage in adipose tissue [104,110].

Although the antioxidant effects of polyphenols are largely attributed to these classical signaling pathways, their regulatory specificity, metabolic stability, and depot-selective actions across different adipose tissues remain insufficiently understood. The pleiotropic and multi-target nature of polyphenols indicates that their benefits are unlikely to arise from a single signaling cascade, but rather from coordinated modulation of multiple interconnected pathways [116]. Future studies should therefore incorporate multi-omics technologies, systems pharmacology, and molecular interaction profiling to systematically compare different polyphenols, identify critical network nodes (such as Nrf2, AMPK, and SIRT1), and elucidate their synergistic mechanisms in regulating adipose tissue redox homeostasis. These insights will be essential for developing polyphenol-based therapeutic strategies targeting oxidative stress in adipose tissue aging.

### 5.2. Suppression of Chronic Inflammation

Polyphenolic compounds exert anti-inflammatory effects through their phenolic hydroxyl groups, which can form hydrogen bonds or π–π interactions with key sites of inflammatory signaling molecules, thereby interfering with the activity and binding processes of inflammation-related enzymes and receptors [117]. At the molecular level, these compounds attenuate proinflammatory signaling primarily by inhibiting NF-κB, MAPK, and JAK/STAT pathways, reducing the transcription and secretion of *TNF-α*, *IL-1β*, and *IL-6* [118]. NF-κB is particularly important in inflammaging due to its dual role in regulating both inflammatory responses and cellular senescence. Under basal conditions, NF-κB remains inactive in the cytoplasm through binding to IκBα. Upon exposure to inflammatory or aging-related stimuli, IκBα undergoes phosphorylation and proteasomal degradation, enabling NF-κB nuclear translocation and transcriptional activation of a wide range of proinflammatory genes, including SASP factors [119,120]. Polyphenols such as curcumin and resveratrol inhibit this process by suppressing IκBα degradation, blocking NF-κB nuclear translocation, and downregulating *TNF-α*, *IL-6*, and *IL-1β* expression, thereby alleviating adipose tissue inflammation and cellular senescence [106,112]. Anthocyanins have also been shown to reduce adipocyte inflammation by inhibiting NF-κB signaling and reduce *IL-6* expression, while improving insulin sensitivity through enhanced PI3K/Akt activation and *GLUT-1* expression [113]. Quercetin has also been shown to improve adipose tissue structure, reduce adipocyte hypertrophy, and decrease the expression of proinflammatory factors such as *Cd11b* and *Cd68* in mice fed a high-fat diet, thereby exerting tissue-specific anti-inflammatory effects [98].

Accumulating evidence confirms that polyphenols such as curcumin [121,122], RES [123,124], anthocyanins [125], and quercetin [96] not only suppress adipose tissue inflammation but also exert broader anti-aging effects in liver, brain, and skeletal muscle, supporting their regulatory role in systemic inflammaging. However, current studies predominantly focus on classical inflammatory pathways, particularly NF-κB, and often overlook broader signaling network interactions, immune cell–adipocyte crosstalk, and depot-specific regulatory differences. Moreover, inflammaging is driven by self-amplifying feedback loops involving immune cell recruitment, SASP propagation, and adipocyte–macrophage interactions, suggesting that targeting a single mediator may be insufficient to disrupt the inflammatory cascade. Future research should therefore focus on identifying key inflammatory amplification nodes, particularly the crosstalk between senescent adipocytes and immune cells, and elucidating how specific polyphenols modulate these networks in a depot- and cell-type–selective manner. Such mechanistic insights will be essential for designing precise, multi-target intervention strategies to mitigate adipose inflammaging.

### 5.3. Regulation of Lipid Metabolism and Promotion of Browning/Thermogenesis

Age-related adipose tissue dysfunction is characterized by impaired lipolysis, excessive lipid accumulation, reduced fatty acid oxidation, and diminished thermogenic capacity, collectively contributing to insulin resistance and metabolic deterioration [108]. Polyphenolic compounds modulate these pathological alterations through multi-target regulation of lipid metabolism, adipogenic transcription networks, thermogenic signaling, and adipokine secretion.

A major mechanism involves the suppression of adipogenesis and lipid storage. Resveratrol inhibits lipid accumulation and differentiation of 3T3-L1 preadipocytes via AMPK activation, leading to the downregulation of lipogenic regulators such as *FAS*, *SREBP-1c*, and *PPARγ* [107,108]. Similarly, EGCG reduces adipocyte differentiation by inhibiting *PPARγ* and *C/EBPα* transcriptional activity and further alleviates lipid accumulation by modulating autophagy-related pathways [100,101,102,103]. These findings suggest that polyphenols reshape the transcriptional landscape of adipocytes, shifting their phenotype away from excessive lipid deposition

Polyphenols also promote lipolysis and enhance the browning and thermogenic conversion of white adipose tissue. Quercetin activates the β3-adrenergic receptor–PKA–AMPK–PGC1-α axis, upregulating UCP1 expression and facilitating the formation of beige adipocytes, which enhances energy expenditure and prevents age-associated adiposity [98,99]. EGCG similarly promotes beige adipocyte formation and improves mitochondrial thermogenic function, thereby counteracting age-related thermogenic decline [126,127].

In addition to metabolic reprogramming, polyphenols help restore endocrine function in aging adipose tissue. Resveratrol reduces leptin secretion while increasing adiponectin levels, thereby improving insulin sensitivity and supporting systemic metabolic homeostasis [114]. Chlorogenic acid enhances adiponectin sensitivity by activating the AMPK pathway, thereby promoting glucose uptake and fatty acid oxidation in skeletal muscle and contributing to the maintenance of metabolic balance [109].

In summary, polyphenols do not merely suppress adipogenesis but instead reprogram adipose tissue from an energy-storing to an energy-expending phenotype through coordinated regulation of AMPK, PPARγ, PGC1α, and UCP1. This multi-pathway modulation offers mechanistic support for their application in mitigating adipose tissue aging and preventing metabolic disorders such as obesity and type 2 diabetes. Future research should focus on identifying tissue-selective regulatory effects and structural determinants of different polyphenols to guide their precise pharmacological use.

## 6. Polysaccharides as Modulators of Adipose Tissue Aging

Natural polysaccharides, primarily derived from medicinal plants, fungi, and edible resources such as *Ganoderma lucidum*, *Lycium barbarum*, and *Lentinula edodes*, have gained increasing interest in adipose tissue aging research due to their unique macromolecular structures, immunomodulatory activity, and high biocompatibility [35]. Similarly to polyphenolic compounds, polysaccharides also demonstrate remarkable value in alleviating adipose tissue aging by scavenging free radicals to counteract oxidative damage, inhibiting the expression of inflammatory factors, and regulating lipid metabolism. To better illustrate their sources, structural characteristics, and key mechanisms involved in adipose tissue aging, representative bioactive polysaccharides are summarized in Table 2.

### 6.1. Mitigation of Oxidative Stress

Oxidative stress and mitochondrial dysfunction are fundamental drivers of adipose tissue aging, and natural polysaccharides mitigate these processes through multiple structural- and signaling-dependent mechanisms. Unlike polyphenols, polysaccharides do not rely on phenolic hydroxyl groups; instead, their antioxidant activities are largely influenced by molecular characteristics such as spatial conformation, molecular weight, degree of branching, and monosaccharide composition. These properties allow polysaccharides to interact with reactive species and transition-metal ions, thereby modulating redox balance at both chemical and cellular levels. Several polysaccharides derived from fungi and plants have demonstrated the ability to directly scavenge superoxide and hydroxyl radicals, stabilize mitochondrial membrane potential, and suppress lipid peroxidation, ultimately preserving mitochondrial integrity and redox homeostasis [128,145,146]. For instance, sulfated fucoidan derivatives chelate Fe^2+^ and Cu^2+^ ions, thereby suppressing hydroxyl radical formation via inhibition of the Fenton reaction and reducing mitochondrial oxidative injury [128]. Similarly, low-methylated pectate forms metal-coordination complexes through its sugar–aldehyde structures, significantly inhibiting lipid peroxidation and protecting mitochondrial function [129]. In addition to direct redox modulation, polysaccharides enhance endogenous antioxidant capacity through activation of the Keap1–Nrf2 signaling pathway. GLP activates the Nrf2/HO-1 axis, increases *SOD* and *GSH-Px* expression, and reduces malondialdehyde (MDA) accumulation [130]. PBRP effectively separates Nrf2 from Keap1, enhancing the activity of antioxidant enzymes such as HO-1, SOD, GSH-Px, and CAT. Additionally, these polysaccharides reduce MDA production by activating the Keap1/Nrf2/HO-1 signaling pathway, demonstrating anti-aging effects [35,132]. The acidic polysaccharide ABM-A from *Agaricus blazei* regulates both Keap1–Nrf2/ARE and MAPK pathways, reducing ROS and lipid peroxidation product MDA levels while enhancing antioxidant enzyme activity, thereby exhibiting significant anti-aging effects in D-galactose–induced aging models [133].

In summary, natural polysaccharides attenuate oxidative stress in adipose tissue through three complementary mechanisms: direct free radical scavenging, metal chelation to inhibit Fenton-driven ROS formation, and activation of endogenous antioxidant signaling pathways—particularly the Keap1–Nrf2/HO-1 axis. The consistent involvement of Nrf2 activation across multiple models suggests that Nrf2 and its downstream effectors may serve as key mechanistic biomarkers for evaluating the anti-aging efficacy of polysaccharides.

### 6.2. Suppression of Chronic Inflammation

Chronic low-grade inflammation driven by SASP factors is a major hallmark of adipose tissue aging and a critical contributor to inflammaging [31,147]. Natural polysaccharides mitigate this process not merely through direct cytokine suppression, but through structure-dependent immunomodulation. Their biological activities are closely associated with molecular weight, monosaccharide composition, sulfation or acetylation levels, and glycosidic bond complexity, which together determine their binding affinity to pattern recognition receptors (PRRs) such as TLR2/4, Dectin-1, and the mannose receptor (MR). These interactions enable polysaccharides to modulate immune–adipocyte crosstalk, influence macrophage polarization, and reshape the adipose immune microenvironment [148,149,150].

At the signaling level, polysaccharides modulate major inflammatory pathways, including TLR4/NF-κB, AMPK/mTOR, SIRT1/NF-κB, and Nrf2/HO-1, thereby downregulating pro-inflammatory mediators (TNF-α, IL-6, IL-1β) and upregulating anti-inflammatory factors (IL-10 and adiponectin). In HFD-induced adipose inflammation, fucoidan markedly reduces TNF-α, IL-1β, and IL-6 expression, suppresses macrophage infiltration (*Cd68*, *Emr1*), while enhancing *Adipoq* and *IL-10*, indicative of restored adipose tissue homeostasis [134]. Similarly, LYTP inhibits the M1 marker *Cd11c* and promotes M2 markers *Arg1* and *Cd206*, facilitating immune microenvironment remodeling toward an anti-inflammatory phenotype [136]. HSP further attenuates adipose tissue inflammation by reducing macrophage activation and indirectly modifying gut microbiota composition, including an increase in *Parabacteroides goldsteinii* [138]. Beyond adipose tissue, polysaccharides also demonstrate systemic anti-inflammatory and anti-aging effects via shared signaling pathways. LBP attenuates hippocampal TNF-α and IL-6 by inhibiting TLR4/NF-κB signaling in OVX-induced aging models, improving neurological function [139]. AMP co-activates AMPK/SIRT1/NF-κB and Nrf2/HO-1 pathways, reducing ROS accumulation and inflammatory stress during brain aging [144]. Moreover, *Astragalus* polysaccharides activate AMPK/mTOR-mediated autophagy, thereby reversing hepatic and adipose aging phenotypes in naturally aged mice [141,142].

Overall, natural polysaccharides alleviate adipose inflammaging not only through inhibition of inflammatory mediators but also by reprogramming the adipose immune–metabolic microenvironment through macrophage polarization, PRR-mediated signaling modulation, gut microbiota interactions, and autophagy regulation. These findings highlight the unique advantage of polysaccharides in coordinating multi-layered inflammatory regulation through both immunological and metabolic pathways. Future studies should focus on elucidating structure–activity relationships and identifying key receptor-mediated signaling hubs to support the precise application of polysaccharides in delaying adipose tissue aging and preventing chronic metabolic diseases.

### 6.3. Regulation of Lipid Metabolism

Age-related adipose dysfunction is closely associated with disturbances in lipid metabolic networks, including enhanced lipogenesis, impaired lipolysis, reduced fatty acid oxidation, and ectopic lipid deposition in non-adipose organs such as the liver. These metabolic abnormalities accelerate insulin resistance, chronic inflammation, and structural degeneration of adipose tissue [131]. Accumulating studies indicate that polysaccharides regulate lipid metabolism through coordinated actions on adipogenesis, lipid catabolism, and mitochondrial fatty acid oxidation.

At the transcriptional level, they inhibit adipogenic programs by downregulating core lipogenic regulators. In 3T3-L1 adipocytes, LBP markedly reduces the expression of *PPARγ*, *C/EBPα*, *FAS*, and *LPL*, thereby suppressing lipid droplet accumulation and early adipocyte differentiation [140]. In vivo, fucoidan decreases *SREBP-1c* and *FASN* expression in HFD-fed mice, alleviating adipocyte hypertrophy and lipid deposition [135]. Polysaccharides from *Ganoderma lucidum* spores similarly suppress *SREBP-1c* and *FASN* expression in MAFLD models, reducing hepatic lipid load and improving systemic metabolic parameters [144]. In parallel with the inhibition of lipogenesis, polysaccharides enhance cellular energy metabolism by activating AMPK-centered signaling pathways. AMPK functions as a metabolic sensor that suppresses ACC activity, relieves inhibition of CPT1, and promotes mitochondrial fatty acid β-oxidation. Its activation also upregulates *PGC-1α* and *PPARα*, further strengthening oxidative catabolism. GLP significantly increases AMPK phosphorylation in HFD mice, accompanied by enhanced ACC phosphorylation, reduced lipid synthesis, and improved hepatic and adipose lipid profiles, ultimately ameliorating insulin resistance [131]. Similarly, LYTP activates AMPK and induces the expression of fatty acid oxidation–related genes, thereby reversing hepatic lipid accumulation and metabolic abnormalities in db/db mice [137].

In summary, natural polysaccharides modulate lipid metabolism through multilayered regulatory actions that include suppressing adipogenic transcriptional programs, inhibiting lipid synthesis, activating the AMPK metabolic hub, and promoting fatty acid oxidation. This combined suppression at the source and enhancement of catabolism framework provides an integrated approach for alleviating lipid metabolic dysfunction in aging adipose tissue. These mechanistic insights underscore the therapeutic relevance of polysaccharides in restoring adipose metabolic resilience and mitigating age-related metabolic disorders.

## 7. Synergistic Mechanisms and Shared Molecular Targets of Polyphenols and Polysaccharides

Although polyphenols and polysaccharides differ substantially in molecular structures and modes of cellular interaction, their regulatory actions on adipose tissue aging converge on several key antioxidant, anti-inflammatory, and metabolic pathways. At the redox level, both compound classes activate the Keap1–Nrf2 axis, thereby inducing downstream antioxidant enzymes such as HO-1 and SOD and enhancing cellular resistance to oxidative stress. In inflammatory regulation, they suppress the activation of NF-κB and reduce the expression of pro-inflammatory cytokines genes including TNF-α and IL-6, attenuating adipose tissue inflammaging. In lipid metabolism, both activate the AMPK signaling pathway, promoting fatty acid oxidation while suppressing lipogenesis, thus contributing to the restoration of metabolic homeostasis. It should also be noted that the beneficial effects of polyphenols and polysaccharides may not be attributed solely to the direct actions of these compounds themselves. Since both compound classes are associated with HO-1 activation, downstream endogenous mediators such as bilirubin may also contribute to the observed antioxidant and metabolic benefits. As a redox-active molecule with reported anti-inflammatory properties and links to PPARα-related signaling, bilirubin may function as a potential synergistic effector in the regulation of adipose tissue aging [151,152].

**Figure 2:**
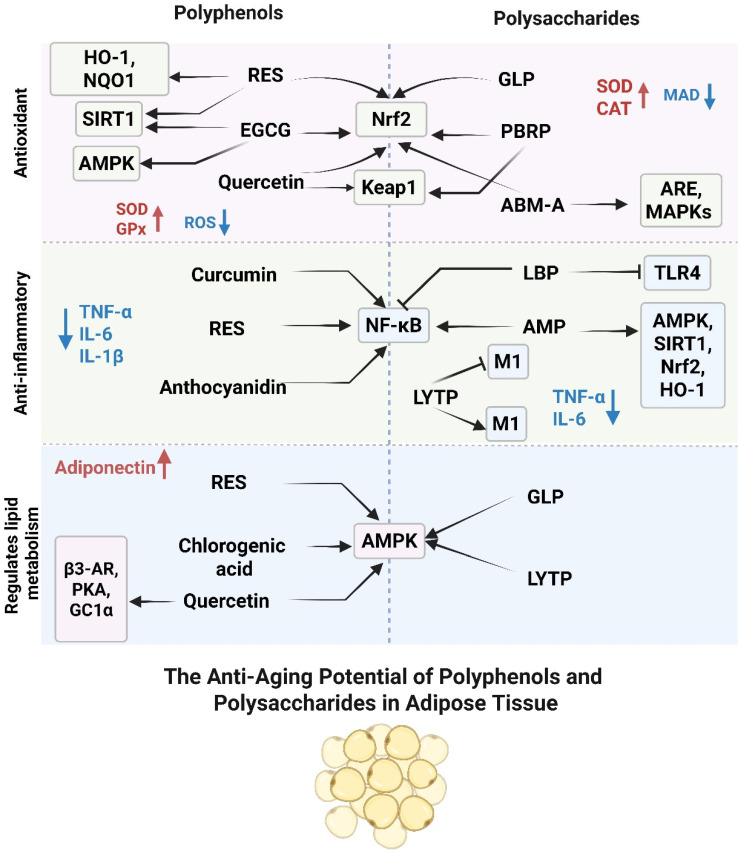
Schematic map of shared molecular targets of polyphenols and polysaccharides in attenuating adipose tissue aging. This diagram depicts the common molecular nodes (Nrf2, NF-κB, and AMPK) and illustrates the potential synergistic interactions through which these bioactive compounds exert antioxidative, anti-inflammatory, and metabolic regulatory effects in senescent adipose tissue. Arrows indicate activation, upregulation, or promotion, whereas blunt-ended lines indicate inhibition or suppression. Red font with upward arrows indicates increased expression or activity, and blue font with downward arrows indicates decreased expression, activity, or accumulation.

These convergent mechanisms suggest that polyphenols and polysaccharides may exert complementary or synergistic effects when used in combination, particularly through coordinated modulation of the Nrf2, NF-κB, and AMPK signaling nodes. To provide a clearer overview of their shared molecular targets and the integrated regulatory network underlying antioxidative, anti-inflammatory, and metabolic actions, the key synergistic pathways are illustrated in Figure 2. However, direct evidence for the combined application of polyphenols and polysaccharides in adipose tissue aging remains very limited. Existing studies have mainly been conducted in obesity-related models rather than in adipose senescence itself. Notably, a mixture of polyphenols and polysaccharides extracted from oolong tea exerted greater beneficial effects on body weight regulation and lipid metabolism than either component used alone in high-fat diet-fed rats [153], suggesting that combination-based strategies may have potential value and warrant further investigation in the context of adipose tissue aging.

## 8. Conclusions and Future Perspectives

Adipose tissue not only maintains systemic metabolic homeostasis but also functions as a central regulator of age-related chronic diseases. Its aging process involves interconnected pathological events, including oxidative stress elevation, chronic low-grade inflammation, and disruptions in lipid metabolic networks. Consequently, targeting adipose tissue aging has become an important strategy for delaying organismal aging and mitigating metabolic disorders. Polyphenolic and polysaccharide natural products have received increasing attention due to their low toxicity, multi-pathway regulatory properties, and broad biological activities. Existing studies demonstrate that polyphenols modulate SIRT1, AMPK, and Nrf2 pathways to reduce oxidative stress, suppress inflammatory signaling, and restore lipid metabolic balance. Polysaccharides, through their macromolecular conformations and immunomodulatory characteristics, activate endogenous antioxidant systems, remodel the adipose immune microenvironment, and enhance lipolysis activity. Together, these findings highlight the potential of these two classes of natural products in regulating adipose tissue senescence. Compared with previous reviews that mainly discuss natural products in the context of general aging, obesity, or metabolic disorders, this review specifically focuses on adipose tissue aging as a distinct biological process and systematically integrates the regulatory roles of both polyphenols and polysaccharides. Importantly, this review not only summarizes their shared anti-aging actions, but also highlights their differential mechanistic characteristics and potential complementarity. Therefore, the present review provides a more targeted framework for understanding and developing natural-product-based interventions for adipose tissue aging.

Beyond these mechanistic findings, the potential therapeutic application of natural products in adipose tissue aging also warrants further consideration. In practical terms, polyphenols may be more relevant in adipose dysfunction characterized by oxidative stress, mitochondrial impairment, and reduced thermogenic activity, whereas polysaccharides may be particularly applicable in settings dominated by chronic low-grade inflammation, immune imbalance, and adipose microenvironment remodeling. Given their distinct biological properties, these two classes of compounds may also offer complementary therapeutic potential. At the same time, the therapeutic value of these agents will depend not only on their intrinsic bioactivity, but also on formulation and delivery strategy. Conventional oral supplementation may be suitable for long-term metabolic support, while nanoformulations and adipose-targeted delivery systems may offer advantages in improving bioavailability, tissue specificity, and overall efficacy. In addition, therapeutic strategies may need to be adapted according to adipose depot characteristics and disease stage, as interventions effective in white adipose tissue dysfunction may not fully address the thermogenic decline associated with brown adipose tissue aging.

Despite these advances, substantial challenges remain in translating natural products into clinically applicable interventions. Polyphenolic compounds such as RES and quercetin exhibit low bioavailability, rapid intestinal metabolism, and limited tissue specificity, restricting their effective in vivo activity [154,155]. Approaches including nano-delivery carriers (liposomes, polymeric nanoparticles), cocrystal engineering, and microbiota-mediated metabolic regulation have been explored to enhance their absorption and stability. However, issues such as low delivery efficiency, inadequate tissue targeting, and inconsistencies between in vitro and in vivo results remain unresolved, and no integrated platform has yet achieved simultaneous high-efficiency loading, controlled release, and adipose-specific targeting [155,156].

Polysaccharides face additional constraints owing to their complex structures and variability between extraction batches. Their biological activities depend on molecular weight, branching degree, and glycosidic linkage features, yet these structural traits are difficult to standardize through natural extraction processes, posing barriers to product development and clinical translation [128,157]. Furthermore, the intestinal absorption and tissue-targeted delivery of polysaccharides remain suboptimal, necessitating strategies such as chemical modification, enzymatic tailoring, and smart delivery systems for improved pharmacokinetic behavior [158]. Future research should therefore focus on optimizing formulation strategies, enhancing bioavailability, advancing targeted delivery technologies, and conducting multi-center clinical evaluations to support their translational progression. Importantly, current evidence supporting the beneficial effects of polyphenols and polysaccharides on adipose tissue aging is still mainly based on in vitro and animal studies, while direct human clinical evidence remains limited. Existing human studies mainly focus on general metabolic outcomes, such as obesity, insulin resistance, or metabolic syndrome, rather than adipose tissue aging–specific endpoints. Therefore, the translational significance of current preclinical findings should be interpreted with caution, and future studies should prioritize well-designed randomized controlled trials with standardized interventions and validated adipose-related biomarkers.

Most current investigations explore polyphenols or polysaccharides in isolation, whereas growing evidence indicates that these compounds exert complementary and synergistic effects. Synergy may arise through the antioxidant, anti-inflammatory, and lipid-regulatory pathways [159,160]. Polyphenols activate the AMPK/SIRT1/Nrf2 axis, providing metabolic and transcriptional support for polysaccharide-mediated immune modulation, such as macrophage polarization. Conversely, polysaccharides regulate gut microbiota composition and intestinal barrier integrity, thereby improving the stability, transformation efficiency, and availability of polyphenols in the gastrointestinal tract. By influencing microbial metabolism and enterohepatic circulation, polysaccharides further enhance polyphenol bioactivation, creating a bidirectional amplification effect [161]. Future research should therefore prioritize rational combination strategies integrating polyphenols and polysaccharides, with systematic evaluation across different adipose depots (WAT and BAT). The development of a “natural-compound–based anti-aging platform” will require the integration of multi-omics approaches—including transcriptomics, metabolomics, and single-cell sequencing—to resolve cellular heterogeneity and map dynamic metabolic pathways. Such comprehensive analyses will facilitate the identification of shared targets, synergistic signaling networks, and spatiotemporal regulatory features underlying their anti-aging effects, ultimately providing a mechanistic foundation for the precise application of natural-compound combinations in adipose tissue rejuvenation.

## Figures and Tables

**Table 1:** Sources and Modes of Action of Polyphenols.

Natural Products	Source	Mode of Action
**Quercetin**	Onions, apples, tea leaves	Antioxidant: Activates the Keap1–Nrf2 pathway, increases the expression of *SOD*, *CAT*, and *HO1*, lowers ROS and MDA levels, and enhances mitochondrial function [96]. Anti-inflammatory: Reduces the expression of *Cd11b* and *Cd68* in adipose tissue, improves tissue structure, and alleviates chronic inflammation [97]. Regulation of lipid metabolism: Activation of β3-AR/PKA/AMPK/PGC1-α promotes the conversion of white fat to brown fat and improves energy metabolism [98,99].
**Epigallocatechin gallate (ECGC)**	Green tea	Antioxidant: Activates the AMPK–SIRT1–Nrf2 pathway, boosts antioxidant enzyme levels, decreases ROS and lipid peroxidation, and enhances mitochondrial metabolism [100]. Regulation of lipid metabolism: Inhibition of *PPARγ* and *C/EBPα* expression, promotion of lipolysis and thermogenesis, and regulation of autophagy [100,101,102,103].
**Resveratrol (RES)**	Grapes, peanuts, mulberries	Antioxidant: Activates the SIRT1 and Nrf2–HO-1–NQO1 pathways, enhances *MnSOD* expression, clears ROS, and alleviates lipid oxidative stress [104,105]. Anti-inflammatory: Inhibits the NF-κB pathway, reduces *TNF-α*, *IL-6*, and *IL-1β* expression, and improves fat inflammation [106]. Regulation of lipid metabolism: activation of AMPK, inhibition of lipid synthesis factors (*FAS*, *PPARγ*), and upregulation of adiponectin [107,108,109].
**Curcumin**	Turmeric	Antioxidant: Increases SOD and GPx activity, inhibits MDA production, activates antioxidant pathways, and improves liver and fat oxidation status [110,111] Anti-inflammatory: Inhibits the nuclear translocation of the NF-κB pathway and prevents the expression of pro-inflammatory factors [112].
**Anthocyanin**	Black rice, berries	Anti-inflammatory: Inhibits NF-κB, reduces IL-6, enhances PI3K/Akt signaling, and improves insulin sensitivity [113].
**Chlorogenic acid**	Coffee, goji berries	Regulation of lipid metabolism: activation of the AMPK pathway, promotion of glucose uptake, and enhancement of adiponectin activity [114].

**Table 2:** Sources and Modes of Action of Polysaccharides.

Natural Products	Source	Mode of Action
**Sulfated fucoidan**	Seaweed (Brown algae)	Antioxidant: Clears hydroxyl radicals, chelates Fe^2+^ and Cu^2+^ to block the Fenton reaction, and reduces mitochondrial oxidative damage [128].
**Pectate**	Fruit peel	Antioxidant: Rich in sugar aldehydes, forming an ‘Egg-box’ shaped complex structure, which reduces lipid peroxidation and helps maintain redox homeostasis [129].
***Ganoderma lucidum* polysaccharides (GLP)**	*Ganoderma lucidum*	Antioxidant: Activates the Nrf2/HO-1 pathway, enhances *SOD* and *GSH-Px* expression, reduces MDA levels, and alleviates oxidative stress [130]. Regulation of lipid metabolism: Activation of AMPK, enhancement of ACC phosphorylation, relief of CPT1 inhibition, promotion of fatty acid entry into the mitochondrial oxidation pathway, reduction in lipid deposition, and improvement of insulin sensitivity [131].
***Phellinus baumii* Residual Polysaccharides (PBRP)**	*Inonotus obliquus*	Antioxidant: Decouples Nrf2 and Keap1, activates the antioxidant enzyme system, and inhibits MDA production [35,132].
**Acidic polysaccharides of ABM-A**	*Agaricus blazei* Murill	Antioxidant: Activates the Keap1-Nrf2/ARE and MAPKs pathways, increases *HO-1*, *SOD*, and *CAT* expression, and inhibits ROS and MDA accumulation [133].
**Fucoidan**	Brown algae	Anti-inflammatory: Inhibits TNF-α, IL-1β, IL-6, reduces *Cd68* and *Emr1* expression, and increases IL-10 [134]. Regulation of lipid metabolism: Inhibition of *SREBP-1c* and *FASN* expression, reducing lipid accumulation [135].
**Large yellow tea polysaccharides (LYTP)**	Chinese rhubarb tea	Anti-inflammatory: Inhibits the M1 macrophage marker *Cd11c* and induces the expression of M2 macrophage markers *Arg1* and *Cd206* [136]. Regulation of lipid metabolism: Activation of AMPK, promotion of fatty acid oxidation, and improvement of metabolic syndrome [137].
***Hirsutella sinensis* polysaccharides (HSP)**	*Hirsutella sinensis*	Anti-inflammatory: Reduces macrophage infiltration, promotes the probiotic *Parabacteroides goldsteinii*, and regulates inflammation [138].
***Lycium barbarum* polysaccharides (LBP)**	Goji berry	Anti-inflammatory: Inhibits the TLR4/NF-κB pathway and reduces *TNF-α* and *IL-6* expression [139]. Regulation of lipid metabolism: Downregulation of *PPARγ*, *FAS*, and *LPL* expression, inhibition of lipid droplet formation [140].
***Astragalus* polysaccharide**	*Astragalus*	Anti-inflammatory: Activates the AMPK/mTOR autophagy pathway to reverse the aging phenotype of adipose tissue [141,142].
***Aronia melanocarpa* polysaccharide (AMP)**	*Aronia melanocarpa*	Anti-inflammatory and antioxidant: Activates AMPK/SIRT1/NF-κB and Nrf2/HO-1 to alleviate inflammation and oxidative stress [143].
***Ganoderma lucidum* Spore Powder**	*Ganoderma lucidum*	Regulation of lipid metabolism: Downregulation of *SREBP-1c* and *FASN* expression improves hepatic lipid accumulation in MAFLD mice [144].

## Data Availability

No new data were created or analyzed in this study. Data sharing is not applicable to this article.
